# Supplementary material for: Exploring the Benefits of Probiotics in Gut Inflammation and Diarrhea—From an Antioxidant Perspective
Source: Antioxidants (Basel). 2023 Jun 26;12(7):1342. doi: 10.3390/antiox12071342 (PMC10376667; doi:10.3390/antiox12071342)
Supplement: Supplementary file 1 [file antioxidants-12-01342-s001.zip › antioxidants-2450887-supplementary.pdf]

Table S1. The alleviating effect of probiotics on IBD.

Microbiology/Dosage	Administration time	Inducer	Subjects	Outcome	Reference
L. plantarum	24 h	TNF-α	Caco-2 cells in vitro	↓TNF-α<br><br>↑SMCT1	[93]
L. plantarum	*24 h*	*Shigella flexneri peptidoglycan-*	*Human monocytic THP-1 cells*	↓ *TNF-α, IL-1β, NF-κB, NOD2*	[94]
L.plantarum MYL26	10 h	LPS	Caco-2 cells in vitro	↓ TOLLIP, SOCS1, and IκBα	[95]
L. plantarum	*24 h*	TNF-α	Caco-2 cells in vitro	↓ TNF-α<br>↑ SMCT1	[96]
L. plantarum Lp62	24 h	*Salmonella enterica serovar Typhi ATCC 6539*	HT-29 cells in vitro	↓ IL-8, TNF-α, IL1- β, and IL-17	[97]
Lactobacillus casei	8 h	*Escherichia coli*	Crohn's disease mucosa.	↓ IL-8 、 IL-6 CXCL1	[98]
Lactobacillus casei strain Shirota	48 h	LPS	Large intestinal lamina propria mononuclear cells	↓ IL-7<br>↓ Nuclear translocation of NF-κB	[99]
L. plantarum Sanriku-SU7<br>*(10^9 CFU/mL)*	4 weeks	Dextran Sodium Sulphate	DSS-induced IBD mice model	Recover colon length	[100]
Lactobacillus plantarum<br>*(2×10^{10} CFU/kg)*	One week	Dextran Sodium Sulphate	DSS-induced colitis mice	↓ Activity of pathogenic bacteria<br><br>Diversified the colon microbes	[101]
Bifidobacterium lactis, Lactobacillus casei and Lactobacillus acidophilus<br><br>*(5×10^8 CFU)*	3 weeks	2,4,6-trinitrobenzene sulfonic acid	TNBS Induced Colitis rat	B. lactis lower diarrhoea rat.<br><br>Bifidobacterium lactis decreased TNF-α, iNOS and COX-2<br><br>L. acidophilus reduced colonic iNOS	[102]
Lactobacillus plantarum LP-Onlly (10^7 CFU/mL)	4 days	Interleukin-10-deficient	IL-10-/- mice	↑ Colon inflammation score	[50]
Lactobacillus plantarum Lp91 (10^9 CFU)	7 days	2,4,6-trinitrobenzene sulfonic acid	TNBS-induced colitis mouse model	↓ TNF-α and COX2 in colitis	[103]

Lactobacillus GG	4 weeks	*Bacteroides vulgatus*	HLA-B27 transgenic rats	Prevented colitis relapse<br>↓ Gross and histological scores<br>↓ MPO, IL-1β, and TNF	[104]
Lactobacillus plantarum 299 V<br>*(10^9 CFU/mL water)*	4 weeks	Interleukin-10-deficient	IL-10-/- mice	↓ Colon mucosal IL-12, IFN-γ	[105]
Lactobacillus reuteri and Lactobacillus fermentum<br>*(5×10^8 colony-forming units/day)*	3 weeks	2,4,6-trinitrobenzene sulfonic acid	TNBS induced murine UC rat model	Reverse the colonic GSH depletion<br>↓ Colonic TNFα levels	[106]
Winbiotic® PRO•IB<br>(7.5×10^9 CFU per day)	Least 6 weeks	Human patients	Mild to moderate UC patients	↑ Quality of life<br>↑ Defecation frequency and stool texture	[107]
Lactobacillus GG<br>(18×10^9 viable bacteria/day)	12 months	Human patients	UC patients with quiescent disease	maintaining remission in UC patients	[108]
Lactobacillus delbruekii and Lactobacillus fermentum (2400 mg/d)	8 weeks	Human patients	Mild to moderate UC patients	↓ Colonic IL-6, TNF-α and NF-κB p65<br>↓ Colonic MPO activity	[44]
Escherichia coli Nissle 1917 (1×2 Kapseln täglich)	Over 1 year	Human patients	UC children	↓ Relapse rate	[109]
Symprove<br>*(1 mL/kg body weight/day)*	4 weeks	Human patients	IBD patients	↓ Faecal calprotectin in the UC patients	[13]

Abbreviate: TNF-α: tumor necrosis factor-α; SMCT1 : sodium-coupled monocarboxylate transporter 1; IL-1β : Interleukin-1β; NF-Kb: nuclearfactorkappa-B; NOD2 : nucleotide binding oligomerization domain containing 2; LPS: lipopolysaccharides; TOLLIP: toll-interacting protein; SOCS1: suppressor of cytokine signaling 1; IκBα: inhibitor kappa B alpha; IL-8: interleukin-8; IL-17: interleukin-17; CXCL1: CXC chemokine ligands 1; IL-6: interleukin-6; IL-7: interleukin-7; TNBS: trinitrobenzenesulfonic acid; iNOS: inducible nitric oxide synthase; COX-2: cyclooxygenase-2; MPO: myeloperoxidase; IFN-γ: interferon-γ

↑ : increase; ↓ : decrease

Table S2. The therapeutic effect of probiotics on intestinal inflammation in weaned piglets.

Item	Strain and dose	Administration time	Intestinal and serum indicators	Others	Reference
Weaned piglets	*Lactobacillus salivarius* (0.5–0.2%)	Starting from weaning and lasting for 14 days	↑ SOD1, GSH-Px4, and CAT in intestine<br>↑ Intestine Claudin-1, Occludin, and ZO-1<br>↓ IL-6β，IL-2，IFN-γ and TNF-α in serum	↑ Average daily gain<br>↓ Diarrhea rate	[9]
Weaned piglets	Mixture of *Lactobacillus acidophilus, Lactobacillus casei, Bifidobacterium thermophilum* and *Enterococcus faecium* (0.1%-0.15%)	Starting from weaning and lasting for 25 days	↓ Serum TNF-α and jejunal MDA<br>↑ Jejunal villus height<br>↑ Jejunal villus height	↑ ADG and ADFI	[11]
Weaned piglets	*Lactobacillus rhamnosus GG*<br>(10^{9} CFU/g LGG.)	Starting from weaning and lasting for 11 days	↑ Jejunal villus height and villus height:<br>↑ mRNA levels of sIgA, ZO-1, occludin and Bcl-2 in jejunal mucosa	Alleviate Diarrhea	[110]
Weaned piglets	Mixture of *B. subtilis, L. sporogenes, B. subtilis var natto* (2g/kg)	Starting from weaning and lasting for 22 days	↑ ileal villus height<br>↓ MDA in the jejunal mucosa<br>↑ Ileum mucosa T-AOC	↓ Diarrhea<br>↓ MDA in the spleen, liver<br>↑ Lactobacillus counts in	[111]

				the ileal digesta	
Piglets	*Bacillus amyloliquefaciens 40* (10^9 CFU/mL/d)	Starting from from birth 6d to 18	↑ serum T-AOC, T-SOD<br>↓ serum IL-1β, INF-γ, MDA, MDA<br>↑ IL-10 and secretory immunoglobulin-A	↓ Diarrhea incidence	[112]
Weaned piglets	*Bacillus licheniformis* (500-1000mg/kg)	Starting from weaning and lasting for 28 days	↑ Serum IgA, IgM, T-AOC, GSH-Px and SOD<br>↓ Serum MDA, IL-1β and IL-6	↓ Diarrhea<br><br>Promote growth	[113]
Piglets	Lactobacillus delbrueckii<br><br>1,2,3, and 4 mL bacterial fluid (50×10^8 CFU/mL) per animal at 1, 3, 7, and 14 d of age	Starting from birth until the age of 49 d	↓ serum diamine oxidase<br>↑ intestinal tight junction proteins	↓ diarrhea<br><br>Promote growth	[114]
Weaned piglets	*Bacillus licheniformis* (500 mg/kg)	Starting from weaning and lasting for 29 days	↑ jejunum mucosal IgA and IgG<br>↓ serum IL-6 and jejunum mucosal IL-1β.	↑ Daily and final weight gain	[115]
Weaned piglets	*Clostridium butyricum* (250-2,000 mg/kg)	Starting from weaning and lasting for 30 days	↑ Jejunal ZO-1<br>↑ Jejunal IL-10	↓ Diarrhea enhancing digestive enzyme activity,	[116]
Weaned piglets	*Lactobacillus plantarum ZLP001* (80 mg/kg)	Starting from weaning	↑ serum SOD, CAT, GSH-PX	↑ Feed conversion rates	[117]

		and lasting for 4 weeks	↓ serum MDA		
Piglets	*Pediococcus acidilactici FT28* (10^9 CFU)	From 7 d of life until 28 d of weaning	↑ serum TAC on 7d and 21d<br>↓ serum on 15d and 21d	↓ Diarrhea	[118]
Weaned piglets	*Bacillus coagulans* (2 × 10^7 CFU/g)	From weaned at 21d and lasting for 21days	↓ Intestine DAO, MDA and H_2O_2<br>↑ Intestine SOD and CAT<br>↑ Villus height in ileum<br>↓ Crypt depth in the jejunum	↑ Intestinal integrity<br>↓ Diarrhea	[81]
Weaned piglets	*B. Licheniformis* (40 g/t)	From weaned at 21d and lasting for 14 days	↑ mRNA expression of SOD1, Nrf2, and HO-1 in the jejunum<br>↑ Jejunum ZO-1<br>↑ Jejunum sIgA levels	↑ Average daily gain<br>↑ Microbial diversity<br>↓ Diarrhea	[119]
Weaned piglets	*Lactobacillus delbrueckii* (0.2%)	Starting from weaning (25 d) and lasting for 28 days	↓ Serum and intestinal mucosa DAO, MDA and 8-OHdG<br>↑ Jejunal GSH-Px, GSH<br>↑ Occludin, ZO-1, and Claudin-1	↑ Antioxidant functions	[120]
Piglets	*Lactobacillus johnsonii BS15* (10^6/CFU/gram of feed)	Starting from birth and lasting for 35 days	↑ CD3CD4/CD3CD8 ratio<br>↓ CD3 and CD8 T cell percentage.	↑ Fecal sIgA<br>↑ Growth performance<br>↓ Diarrhea<br>↓ Escherichia coli	[67]

				populations on day 35 of treatment.	

Abbreviate: SOD1: superoxide dismutase; GSH-Px4: glutathione peroxidase; CAT: catalase; ZO-1: zonula occludens protein 1; IL-6β: interleukin-6; IL-2: interleukin-2; IFN-γ :interferon-γ; TNF-α: tumor necrosis factor-α; ADG :average day gain; ADFI: average daily feed intake; sIgA: secretory immunoglobulin A; Bcl-2: B-cell lymphoma-2; MDA: malondialdehyde; T-AOC: total antioxidant capacity; IL-10: interleukin 10; TAC:total antioxidant capacity; Nrf2: nuclear factor erythroid2-related factor 2; DAO: diamine oxidase; 8-OHdG: 8-hydroxy-2' -deoxyguanosine; CD3/4/8: cluster of differentiation 3/4/8; ↑： increase; ↓： decrease
